# Editorial: Mitral valve disease: mechanisms to therapies

**DOI:** 10.3389/fcvm.2024.1441511

**Published:** 2024-06-18

**Authors:** Maria Carmo Pereira Nunes, Hoda Hatoum, Alison M. Pouch, Muralidhar Padala

**Affiliations:** ^1^ Department of Internal Medicine, Federal University of Minas Gerais, Belo Horizonte, Brazil; ^2^ Department of Biomedical Engineering, Michigan Technological University, Houghton, MI, United States; ^3^ Health Research Institute, Center of Biocomputing and Digital Health and Institute of Computing and Cybersystems, Michigan Technological University, Houghton, MI, United States; ^4^ Department of Radiology, University of Pennsylvania, Philadelphia, PA, United States; ^5^ Department of Bioengineering, University of Pennsylvania, Philadelphia, PA, United States; ^6^ Department of Surgery, Emory University, Atlanta, GA, United States

**Keywords:** mitral valve disease, mitral regurgitation, mitral valve repair, outcome, atrial fibillation

**Editorial on the Research Topic** Mitral valve disease: mechanisms to therapies

Mitral valve disease (MVD) represents a complex spectrum of conditions with significant implications for cardiovascular outcomes. Advances in our understanding of the structural mechanisms underlying MVD have paved the way for innovative therapeutic strategies. The current Research Topic delves into various aspects of mitral valve regurgitation (MR), including its mechanisms, progression, and treatment, with a particular focus on transcatheter edge-to-edge repair (TEER). This innovative, minimally invasive procedure is especially beneficial for patients at high surgical risk due to advanced age, comorbidities, or other factors. TEER has shown promising results in reducing symptoms, improving quality of life, and decreasing hospitalizations related to heart failure, making it a valuable option in the management of MR.

MR is one of the most prevalent valvular heart diseases (VHD) globally, playing a significant role in the development of heart failure (HF). Atrial fibrillation (AF) emerges as the most frequent condition associated with MR and is closely linked to a poor prognosis. Given the high perioperative risk in an aging population burdened by multiple comorbidities, TEER is an effective treatment modality, offering relief from HF symptoms and improving prognosis in select MR etiologies.

Addressing the impact of AF treatment on outcomes following TEER in this current research topic, Ausbuettel et al. conducted an analysis of 821 patients undergoing TEER. Among them, 608 patients (74.1%) had AF, with 48 individuals undergoing catheter ablation (CA). Patients with AF who underwent CA showed significantly higher 3-year survival rates after TEER compared to those on pharmacological AF treatment, and their survival rates were similar to those of patients without AF. This study underscores the importance of treating both MR and concomitant AF in this high-risk population.

MR due to flail leaflet is associated with a high risk of morbidity and mortality, primarily resulting from left ventricular dysfunction, heart failure, and death. Although mitral valve surgery has traditionally been the standard of care for patients with flail leaflets, TEER is now emerging as a safe and effective alternative, particularly for high-risk patients who may not be suitable candidates for surgery. In another study from this research topic focusing on high-risk surgical patients, Perel et al. assessed individuals with degenerative mitral valve disease presenting with ruptured chordae necessitating TEER. Among the 49 patients included, 17 (35%) underwent urgent intervention. The study found no significant difference in overall mortality between those who received urgent versus elective TEER. Additionally, postprocedural hemodynamic and echocardiographic parameters demonstrated significant improvement in the severity of MR.

Secondary MR can also develop in response to left-sided chamber remodeling triggered by conditions such as aortic insufficiency (AI). In a complementary investigation within this research topic, Xu et al. assessed MR progression in patients with significant AI undergoing aortic valve intervention. Among 347 patients, 10 (3.6%) experienced MR deterioration, with preoperative AF and AI due to rheumatic heart disease identified as independent risk factors. These findings highlight the need for follow-up and consideration of concomitant valve lesions in MR management.

Lastly, Molisana et al. reviewed the mechanisms of MR in hypertrophic cardiomyopathy (HCM), illustrating two different MR mechanisms through a case report. In obstructive HCM, systolic anterior motion-dependent MR occurs due to drag forces attracting the anterior mitral leaflet during systole, causing eccentric regurgitation. Additionally, intrinsic abnormalities of the mitral valve apparatus, independent of left ventricular outflow tract obstruction, can lead to MR. These abnormalities include leaflet elongation, papillary muscle anomalies, annular geometry changes, and chordal rupture with flail leaflets. Despite these insights, the mechanisms leading to MR in HCM are not fully understood and warrant further investigation.

In conclusion, this research topic underscores the importance of a multifaceted approach to MVD, encompassing both advanced therapeutic strategies and a thorough understanding of underlying mechanisms to improve patient outcomes.

